# Family characteristics and loneliness among older adults: evidence from the Brazilian Longitudinal Study of Aging (ELSI-Brazil)

**DOI:** 10.1590/1980-549720240054

**Published:** 2024-11-22

**Authors:** Karla Geovani Silva Marcelino, Luciana de Souza Braga, Maria Fernanda Lima-Costa, Juliana Lustosa Torres

**Affiliations:** I Universidade Federal de Minas Gerais – Belo Horizonte (MG), Brazil.; II Fundação Oswaldo Cruz, Center for Studies in Public Health and Aging – Belo Horizonte (MG), Brazil.

**Keywords:** Residence characteristics, Family characteristics, Loneliness, Aging, Health of the elderly

## Abstract

**Objective:**

To investigate the association between family characteristics concerning partners and children and loneliness among Brazilians aged 50 and over, taking into account both the occurrence of loneliness and its severity levels.

**Methods:**

This cross-sectional study used data from 7,163 participants in the second wave of the Brazilian Longitudinal Study of Aging, a nationally representative study conducted in 2019-2021. Loneliness was assessed using the 3-item University of California Loneliness Scale. Family characteristics included: marital status and living with the partner and presence of children and living with them. Statistical analysis employed Zero-Inflated Negative Binomial regression models, allowing the assessment of the outcome in both dichotomous and counting-based forms.

**Results:**

Only family characteristics related to the partner were associated with loneliness prevalence, whether in a living-apart-together arrangement (PR=0.35; 95%CI 0.23–0.53) or cohabiting (PR=0.37; 95%CI 0.30–0.45). Family characteristics concerning the partner [cohabiting (PR=0.80; 95% CI 0.73–0.88)] and children [non-cohabiting (PR=0.86; 95%CI 0.77–0.95) or cohabiting (PR=0.81; 95%CI 0.72–0,92)] were negatively associated with loneliness levels.

**Conclusion:**

Family characteristics play a crucial role in both preventing loneliness and reducing its levels. Public services for improving social support should target older adults with reduced nuclear families.

## INTRODUCTION

The need for connection is a fundamental human trait, manifested through relationships and feelings of companionship. In this context, loneliness arises when this relational need goes unmet, resulting in an unpleasant emotional state^1,2^. This differs from objective measures of social isolation^2^. Although the definition of loneliness lacks consensus in the literature, it is well established that it can have negative effects on both mental and physical health^2^, including cognitive decline^3,4^, an increased risk of suicide attempts^5^, and a higher likelihood of cardiovascular disease^6^.

Furthermore, loneliness is regarded as an epidemic^7^, with a prevalence of 13.2% (95% confidence interval — 95%CI 9.2–18.6) among individuals aged 60 years or older, according to data from a meta-analysis^8^. Older adults without an intimate partner, those who have recently lost a partner, those with a limited social network and/or low levels of social activity, and those experiencing depression are at a higher risk of loneliness^9,10^.

Courtin and Knapp^11^ explain that both loneliness and social isolation become more problematic in aging due to the reduction of economic and social resources, functional limitations, widowhood, and changes in family structures. The family is a cultural construct and a social institution composed of at least two individuals connected by ties of kinship, adoption, or marriage, and the way it is organized within the home is referred to as the household arrangement^12^.

In terms of living arrangements, residing with a spouse and/or children can provide older adults with greater perceived or actual social, emotional, and instrumental support^13^, which may help reduce loneliness. A longitudinal study conducted in China among individuals aged 65 and older found that the incidence of loneliness was similar between those living with their children and those living only with their spouse but was lower compared to those living alone^13^. Among older Dutch and Italian adults aged 55 to 89, living with a spouse, regardless of whether children were present in the household, was associated with lower levels of loneliness^14^. Additionally, a cross-sectional study of octogenarians in Germany found that having a spouse living in a separate household was linked to increased loneliness^10^.

Among older Brazilian adults aged 50 and over, living alone^15,16^ or with two people^16^ has been associated with loneliness. However, no Brazilian studies were found that examined the association between family characteristics (such as having a spouse and/or children and living with them) and loneliness. In Brazil, the family is culturally and legally viewed as the primary entity responsible for the social, emotional, and economic well-being of its members^17^, which may differ from the cultural context of other countries and suggest a distinct role in relation to loneliness^18,19^. Therefore, the aim of the present study was to investigate the association between family characteristics [presence of a spouse or children and cohabitation with them] and loneliness in Brazilians aged 50 and over, considering both the occurrence of loneliness and its varying levels.

## METHODS

This cross-sectional study is based on data from wave 2 of the Brazilian Longitudinal Study of Aging (*Estudo Longitudinal da Saúde dos Idosos Brasileiros* – ELSI-Brazil), conducted between 2019 and 2021. ELSI-Brazil is a nationally representative study of the Brazilian population aged 50 years old and older living in the community, utilizing a probability sample. The sample was designed in three stages of selection (municipalities, census tracts, and households) and includes 70 municipalities from the five major geographic regions of the country^20^. In selected households, all residents aged 50 and over were eligible to participate. The questionnaire consists of a household module, completed by a resident capable of providing information about the household's characteristics and the socioeconomic details of its residents, and an individual module, answered by the participant or a proxy respondent.

ELSI-Brazil began in 2015, and subsequent waves are conducted every three years, with sample replacements. In wave 2, used for the present analysis, 9,949 participants were included, with a response rate of 75.9% compared to the previous wave. The combination of sample replacement and the addition of new participants over time is essential to maintaining the national representativeness of the study^20,21^. Further details are available in other publications and on the study's official website (https://elsi.cpqrr.fiocruz.br/).

ELSI-Brazil was approved by the Research Ethics Committee of Fundação Oswaldo Cruz (FIOCRUZ), Minas Gerais, Brazil (protocol No. 34649814.3.0000.5091). Participants provided written informed consent for each of the research procedures.

**Figure 1:**
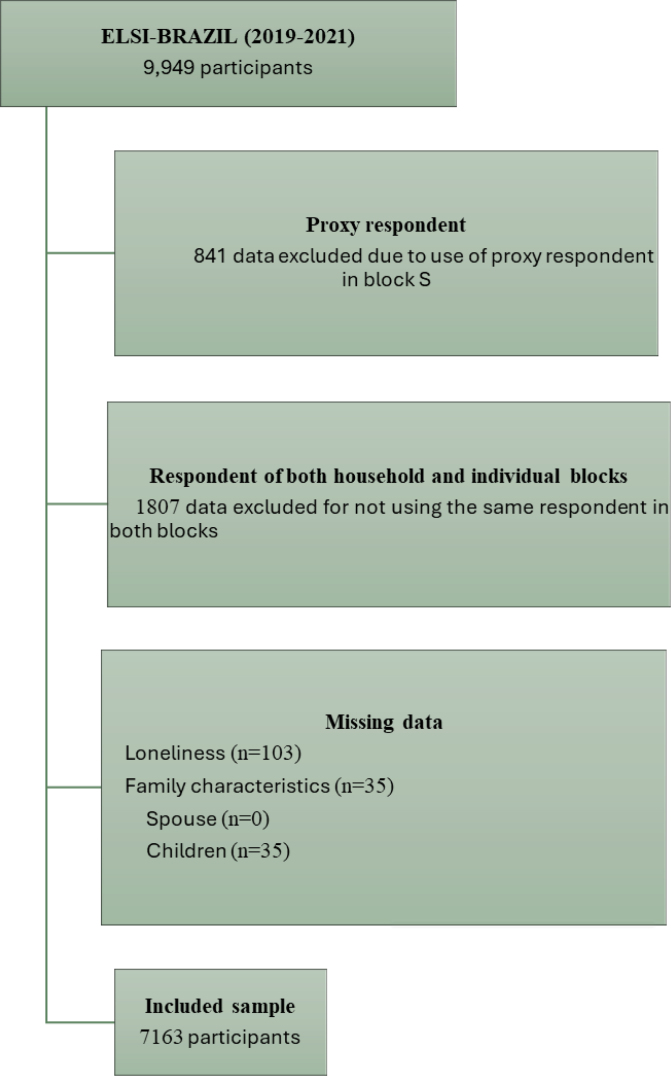
Flowchart of the participants included in the study. Brazilian Longitudinal Study of Aging (ELSI-Brazil), 2019–2021.

Since loneliness is a subjective experience, dependent on an individual's personal perception^21^, only the 9,108 participants who directly responded to the psychosocial section of the interview (*i.e.*, without the use of a proxy respondent in block S) were eligible for the present analysis. From this group, only participants who completed both the household and individual modules (n=7,301) were included. Additionally, participants with missing data on the loneliness variable (n=103) and family characteristics related to the spouse (n=0) and children (n=35) were excluded. As a result, the final sample for this study consisted of 7,163 participants, as shown in Figure 1.

Loneliness was assessed using the three-item University of California, Los Angeles (UCLA) Loneliness Scale^22^, which includes three questions related to feelings of lacking companionship, feeling left out, and feeling isolated from others. Response options (hardly ever, some of the time, or often) produced a final score ranging from 3 (no loneliness) to 9 points, with higher scores indicating greater loneliness. The long version of the scale was translated and validated for the Brazilian elderly population, yielding a Cronbach's alpha of 0.88^23^. The three items on the shortened scale showed correlations close to 0.60 with the total score (lack of companionship [r=0.60], feeling left out [r=0.54], and feeling isolated from others [r=0.61])^23^.

The independent variables consisted of two family-related characteristics. The first was the presence of a spouse, obtained from the individual module, and cohabitation with the spouse, obtained from the household module. Similarly, the second family characteristic was related to the presence of children and cohabitation with them.

Family characteristics related to the spouse were categorized based on marital status and living arrangements. Participants who had a partner (married, cohabiting, or in a stable union) living in the same household were classified under the "spouse living in the same household" group. Those who reported having a partner but indicated that the partner lived elsewhere were placed in the "spouse living in another household" group. The remaining participants, single, divorced, or widowed, were classified under the "no spouse" group.

Family characteristics related to children were categorized based on the presence of living children and their living arrangements. Participants with at least one living child but none residing in the household were classified under the "children living in another household" group. Those with at least one living child and at least one residing in the household were included in the "children living in the same household" group. Participants who reported having no living children were placed in the "no children" group.

Sociodemographic and health characteristics were considered potential confounding variables, consistent with the literature on risk factors for loneliness^9^, which may reflect changes in family characteristics. The sociodemographic variables included sex (female or male); age range (50–59; 60–69; 70–79; and ≥80 years); and per capita household income categorized into tertiles (lower tertile, intermediate tertile, and upper tertile). The health characteristics assessed were cognitive impairment (no or yes), depression (no or yes), and limitations in basic activities of daily living (ADLs) (no or yes). Cognitive impairment was evaluated using cognitive tests similar to those employed in the Health and Retirement Study^24^:

- temporal orientation regarding the day, month, year, and day of the week of the interview, generating one point for each correct response;
- immediate and delayed recall of ten words, where participants were required to repeat ten words immediately after reading them (immediate memory) and to repeat the same words after 5 minutes (delayed memory). Scores ranging from 0 to 10 were calculated for the number of words recalled in the immediate and delayed recall tests, separately;
- semantic verbal fluency, assessed by the number of different animals recalled in up to 1 minute.

The results of each test were converted to z-scores by subtracting each participant's score from the mean score of all participants and dividing by the standard deviation (SD). A single score was generated by averaging the z-scores of all tests. The cutoff point used to define cognitive impairment was a global cognitive z-score of equal to or less than −1 SD^25^.

Depression was assessed through self-reporting of a medical diagnosis. Limitations in ADLs were considered when participants reported difficulty in at least one of the following activities: walking from one room to another, getting dressed, bathing, using the bathroom, eating, or lying down in bed/getting out of bed.

For the statistical analysis, the variables were initially described for the total sample and according to levels of loneliness, using proportions and means. Differences observed between proportions were analyzed using Pearson's χ^2^ test with Rao-Scott correction, while differences between means were assessed using the Wald test or paired t-test. Subsequently, zero-inflated negative binomial regression models were constructed to estimate prevalence ratios (PR) and 95%CI for the association between family characteristics and the loneliness score. This model combines an excess of zeros model (absence *versus* presence of loneliness; score 3 *versus* ≥4), which estimates the PR for the absence of loneliness, with a negative binomial counting model (level of loneliness; scores ranging from 4 to 9)^26^. This approach was appropriate since the loneliness score (dependent variable) exhibited overdispersion (α=0.03; p<0.01) and a high number of individuals without loneliness were observed (score 3; 56%)^27^.

The zero-inflated negative binomial model accounts for the zero score (equivalent to a loneliness score of 3) as resulting from two types of individuals: those who consistently report the absence of loneliness ("structural zeros") and those who experience loneliness but did not report it during the study period ("sample zeros")^27^. To enable the model to treat a score of 3 as zero, the loneliness scores were transformed to a range from 0 to 6. However, the authors opted to retain the original scores (3 to 9) throughout the text, as these values are commonly reported in the literature. To facilitate the interpretation of the PRs in the model that accounts for excess zeros, the coefficients were inverted to reflect the PR of the prevalence of loneliness (score 3 *versus* ≥4) rather than the absence of loneliness (excess zeros).

All analyses were conducted using Stata/Se software (StataCorp., College Station, TX, United States), version 17.0, taking into account the sampling design and participant weights.

## RESULTS

Among the 7,163 participants included in the study, mean age was 63.1 years (SD = 8.8 years). Of these participants, 44.1% were classified as experiencing mild loneliness, with a mean loneliness score of 5.2 (SD = 1.2) and a median score of 5 (interquartile range of 4 to 6) when loneliness was present. Since the mean and median scores were similar, and the mean was more effective in highlighting differences between the analyzed groups, the means were used to describe the variables.

Table 1 presents the characteristics of the participants, both in total and according to the proportion and mean of the loneliness score. The majority of participants were female (52.5%) and aged up to 69 years (77.6%). Additionally, 54% lived with a spouse in the same household, and 58% had children living in another household. The family characteristics varied according to the prevalence of loneliness (score ≥4) only when considering the spouse (p<0.001). However, the means of the loneliness level (scores from 4 to 9) differed both between the family characteristics related to the spouse and those related to the children, being lower in the categories of spouse living in the same household (p<0.001), children living in another household (p = 0.006), and children living in the same household (p<0.001).

**Table 1:** Distribution of sociodemographic, health, and family characteristics of participants, total and by level of loneliness (Brazilian Longitudinal Study of Aging, 2019–2021).

Characteristics		Total (%)	Loneliness		
			No loneliness (score 3) (% and 95%CI)*	Presence of loneliness (score ≥4) (% and 95%CI)*	Level of loneliness (score 4 to 9) (mean and SD)†
Sociodemographic					
Sex					
	Female	52.5	**49.1 (45.0–53.2)**	**50.9 (46.8–55.0)**	5.3 (1.3)
	Male	47.5	**63.3 (58.8–67.5)**	**36.7 (32.5–41.2)**	5.2 (1.1)
Age range (years)					
	50–59	47.7	55.6 (58.9–60.2)	44.4 (39.8–49.1)	**5.3 (1.0)**
	60–69	29.9	57.0 (53.2–60.8)	43.0 (39.2–46.8)	**5.1 (1.3)**
	70–79	16.1	56.5 (52.2–60.7)	43.5 (39.3–47.8)	**5.2 (1.5)**
	≥80	6.3	50.6 (43.8–57.5)	49.4 (42.5–56.2)	**5.3 (1.5)**
Household income per capita					
	Lower tercile	33.4	54.2 (48.6–59.8)	45.8 (40.2–51.4)	**5.4 (1.2)**
	Intermediate tercile	33.3	53.5 (49.0–58.0)	46.5 (42.0–51.0)	**5.2 (1.3)**
	Upper tercile	33.3	59.1 (54.9–63.2)	40.9 (36.8–45.1)	**5.1 (1.2)**
Health					
Cognitive impairment‡					
	No	78.4	**57.5 (53.4–61.6)**	**42.5 (38.4-46.6)**	**5.1 (1.2)**
	Yes	21.6	**52.1 (47.5–56.6)**	**47.9 (43.4–52.5)**	**5.4 (1.4)**
Depression					
	No	87.3	**58.7 (54.6–62.7)**	**41.3 (37.3–45.4)**	**5.1 (1.2)**
	Yes	12.7	**36.4 (31.1–42.1)**	**63.6 (57.9–68.9)**	**5.8 (1.4)**
Limitations in ADLs§					
	No	91.9	**57.6 (53.7–61.4)**	**42.4 (38.6–46.3)**	**5.2 (1.2)**
	Yes	8.1	**35.9 (30.0–42.3)**	**64.1 (57.7–70.0)**	**5.6 (1.3)**
Family characteristics					
Spouse					
	No spouse	42.0	**42.1 (38.4–45.9)**	**57.9 (54.1–61.6)**	**5.4 (1.4)**
	Spouse living in another household	4.1	**63.2 (53.7–71.8)**	**36.8 (28.2–46.3)**	**5.4 (1.2)**
	Spouse living in the same household	53.9	**66.0 (61.2–70.5)**	**34.0 (29.5–38.8)**	**5.0 (1.0)**
Children					
	No children	9.1	50.2 (42.5–57.9)	49.8 (42.1–57.5)	**5.5 (1.1)**
	Children living in another household	57.9	55.5 (52.0–59.0)	44.5 (41.0–48.0)	**5.2 (1.3)**
	Children living in the same household	33.0	57.9 (52.1–63.5)	42.1 (36.5–47.9)	**5.1 (1.2)**
**N total**		7,163	3,823	2,897	443

* values in bold: p<0.05 based on the Pearson χ^2^ test with Rao-Scott correction;

† values in bold: p<0.05 based on the Wald test or paired t-test;

‡ considering temporal orientation, immediate and delayed memories, and semantic verbal fluency;

§ difficulty in walking from one room to another, dressing, bathing, using the bathroom, eating, or lying down/getting out of bed. 95%CI: 95% confidence interval; SD: standard deviation; ADLs: basic activities of daily living.

Table 2 outlines the association between the prevalence/level of loneliness and various sociodemographic, health, and family characteristics. Sociodemographic characteristics (male sex) were associated with a lower prevalence of loneliness, while health characteristics (depression and limitations in ADLs), were linked to a higher prevalence of loneliness. In terms of family characteristics, the prevalence of loneliness was 65% lower among individuals who had a spouse living in another household (PR=0.35; 95%CI 0.23–0.53) and 63% lower among older adults whose spouses lived in the same household (PR=0.37; 95%CI 0.30–0.45), compared to those without a spouse.

**Table 2:** Results of crude and adjusted models for the association between family characteristics and loneliness, adjusted for sociodemographic and health characteristics (n=6,063) (Brazilian Longitudinal Study of Aging, 2019–2021)*.

Characteristics		Presence of loneliness (score 3 versus ≥4)				Level of loneliness (increase of one unit in score from 4 to 9)			
		Crude Models		Adjusted Model		Crude Models		Adjusted Model	
		PR†	95%CI	PR†	95%CI	PR	95%CI	PR	95%CI
Sociodemographic characteristics									
Male sex (*versus* female)		**0.56**	**0.48–0.65**	**0.75**	**0.61–0.92**	0.95	0.88-1.02	1.02	0.93–1.11
Age range (*versus* 50–59 years)									
	60–69	0.96	0.81–1.12	0.92	0.74–1.14	**0.89**	**0.83–0.97**	0.91	0.84–1.00
	70–79	0.98	0.81–1.18	0.83	0.63–1.07	0.93	0.85–1.02	**0.88**	**0.87–0.97**
	≥80	1.17	0.91–1.51	0.95	0.64–1.40	1.02	0.92–1.13	0.96	0.82–1.12
Household income per capita (*versus* lower tertile)									
	Intermediate tertile	1.09	0.89–1.34	1.06	0.83–1.36	0.93	0.84–1.02	**0.90**	**0.81–0.99**
	Upper tertile	0.88	0.72–1.08	0.80	0.62–1.01	**0.86**	**0.78–0.95**	**0.82**	**0.74–0.91**
Health Characteristics									
	Cognitive impairment (*versus* no)	**1.13**	**1.05–1.23**	1.00	0.80–1.25	1.16	0.98–1.38	1.07	0.98–1.17
	Depression (*versus* no)	**2.17**	**1.76–2.67**	**2.05**	**1.52–2.77**	**1.44**	**1.34–1.56**	**1.42**	**1.28–1.56**
	Limitations in ADLs (*versus* no)	**2.44**	**1.87–3.20**	**1.92**	**1.31–2.80**	**1.27**	**1.16–1.39**	**1.18**	**1.04–1.34**
Family characteristics									
Spouse (*versus* no spouse)									
	Spouse living in another household	**0.36**	**0.26–0.51**	**0.35**	**0.23–0.53**	1.01	0.87–1.17	1.01	0.84–1.22
	Spouse living in the same household	**0.39**	**0.33–0.45**	**0.37**	**0.30–0.45**	**0.83**	**0.77–0.89**	**0.80**	**0.73–0.88**
Children [*versus* no children]									
	Children living in another household	0.87	0.67–1.12	1.29	0.92–1.81	**0.86**	**0.78–0.95**	**0.86**	**0.77–0.95**
	Children living in the same household	0.84	0.63–1.13	1.18	0.81–1.73	**0.80**	**0.71–0.90**	**0.81**	**0.72–0.92**

ADLs: basic activities of daily living; PR: prevalence ratio; 95%CI: 95% confidence interval;

* values in bold: p<0.05 based on zero-inflated negative binomial regression;

† PR reversed to reflect the presence of loneliness (score ≥4) rather than the absence of loneliness (score=3).

Table 2 indicates that sociodemographic characteristics (age group [70–79 years] and per capita household income [middle and upper tertile]) were associated with lower levels of loneliness. In contrast, health characteristics (depression and limitations in ADLs) were linked to higher levels of loneliness. Regarding family characteristics, the level of loneliness was, on average, 20% lower among older adults whose spouses lived in the same household (PR=0.80; 95%CI 0.73–0.88), compared to individuals without a spouse. This association was not observed for older adults whose spouses lived in another household (PR=1.01; 95%CI 0.84–1.22). Concerning children, the level of loneliness was, on average, 14% lower among those whose children lived in another household (PR=0.86; 95%CI 0.77–0.95) and 19% lower among those whose children lived in the same household (PR=0.81; 95%CI 0.72–0.92), compared to individuals without children.

## DISCUSSION

This study demonstrated that family characteristics were significantly associated with both the prevalence and the severity of loneliness. Specifically, characteristics related to the spouse were linked to lower prevalence rates of loneliness (individuals with a spouse living in the same or another household experienced reduced feelings of loneliness). Conversely, the presence of children, regardless of whether they lived in the same household, did not show a significant impact on the prevalence of loneliness. Moreover, once loneliness was experienced, family characteristics concerning both spouses and children were inversely associated with higher levels of loneliness. Notably, older adults who lived with a spouse in the same household exhibited lower levels of loneliness, as did those with children, whether residing with them or not.

Our findings align with the existing literature indicating that the highest prevalence of loneliness is observed among older adults without a spouse^9,10,28–30^. These results can be elucidated by the socioemotional selectivity theory proposed by Carstensen^31^, which suggests that the socioemotional losses associated with aging are mitigated through emotional self-regulation. In this context, socioemotional resources are redistributed as the perspective on future time changes, leading to a greater emphasis on close relationships, which become more fulfilling. Additionally, the cultural influence on the experience of loneliness may play a significant role, as it moderates the effect of various relationship types on feelings of loneliness. This is supported by the study conducted by Lykes and Kemmelmeier, which highlights the importance of belonging in collectivist European countries, where individuals place significant value on family relationships and emotional connections^19^.

In the ELSI-Brazil sample, a prior cross-sectional study indicated that individuals living in housing arrangements of two or three people or more exhibited nearly identical prevalence rates of loneliness at different times (31.1 and 32.5, respectively^16^. It is well-established that cohabitation with a spouse remains the most common type of living arrangement in Brazil, despite the emergence of new configurations in living arrangements^32^.

The spouse often serves as the primary source of positive emotional social support^33^, which can significantly alleviate stressors linked to poorer mental health^34^. This support also enhances opportunities for engaging in partner-related activities, thereby promoting social participation and the establishment of connections^35^. A longitudinal study conducted with Mexicans aged 50 years old and older found that individuals who perceived support from their spouse experienced lower levels of loneliness after three years^36^.

In the context of family characteristics, this study found that having a spouse living in another household was not linked to a higher level of loneliness, contrasting with earlier findings among octogenarians in Germany^10^. This discrepancy may arise for several reasons. Firstly, having a spouse living apart could signify a second union or reflect negative experiences with a previous spouse or even institutionalization^10,37^. Secondly, a spouse can still provide social support despite physical distance^19^, suggesting that balance and reciprocity in social interactions are crucial^38^. Living in separate households may facilitate the preservation of this balance and reciprocity within the relationship. However, the existing literature on the implications of having a spouse in another household remains limited, complicating the interpretation of these findings.

Concerning children, the findings of this study indicate that having children residing in the same household or in separate households decreases levels of loneliness. A potential explanation for the reduced loneliness experienced by those with children could be, in addition to the expanded family network, the variety of interactions with children, grandchildren, and other relatives^10^, regardless of any geographical distance. This connection may be facilitated through both virtual and face-to-face interactions.

In Brazil, face-to-face or virtual contact with children residing in separate households is quite common among older adults^39^, serving as a significant source of emotional and instrumental social support in times of need^39,40^. In Japan, a longitudinal study involving individuals aged 65 and older discovered that those living with at least one child had a lower probability of reporting loneliness compared to those without children in their household^41^. Furthermore, among individuals without any children living with them, a greater number of children was associated with a reduced likelihood of reporting loneliness^41^.

In a study conducted by Zoutewelle-Terovan and Liefbroer involving a representative sample of individuals aged 50 to 85 from 12 European nations, higher levels of loneliness were observed among those without children, with the magnitude of this effect varying according to the cultural values of each country^42^. The presence of children can foster a sense of mutual security among individuals, particularly in collectivist societies where family relationships are essential to well-being^19^.

The literature suggests that individuals without children often diversify their sources of social support, similar to couples with low mutual social support^43^, which may help mitigate feelings of loneliness. Nevertheless, health and social assistance professionals should remain vigilant regarding the emotions of older adults and the characteristics of their families, aiming to foster meaningful relationships and to prevent and reduce the risk of loneliness.

This study has both strengths and weaknesses. One notable strength is its pioneering approach to analyzing family characteristics related to marital status, the presence of children, and cohabitation, as well as their association with loneliness in a representative sample of older Brazilians. Additionally, the application of the zero-inflated negative binomial model in the statistical analyses enables differentiation between factors associated with the prevalence of loneliness and its intensity. However, the scale used to assess loneliness, while widely adopted internationally, has not been validated in its shortened form for the Brazilian population. Furthermore, the cross-sectional design of the study prevents the establishment of causal relationships between family characteristics and loneliness. Future research could explore the directionality of the observed associations. It is important to note that ELSI-Brazil is a prospective cohort study, and forthcoming longitudinal analyses could examine the temporality of the associations identified in this study.

Furthermore, it is crucial to emphasize that family characteristics play a significant role in both preventing loneliness and reducing its severity. Consequently, assistance efforts should target older adults who receive less family support, such as those without a spouse or children. It is suggested that investments be made in public services designed to meet the specific needs of individuals, ensuring that greater social support is provided by the State to those with smaller family groups. Loneliness is a detrimental emotional state regarded as a public health issue and is potentially modifiable, making its screening essential, particularly among individuals at higher risk.
