# Supplementary material for: Family characteristics and loneliness among older adults: evidence from the Brazilian Longitudinal Study of Aging (ELSI-Brazil)
Source: Rev Bras Epidemiol. 2024 Nov 22;27:e240054. doi: 10.1590/1980-549720240054 (PMC11654287; doi:10.1590/1980-549720240054)
Supplement: Translation

# Translation: Características da família e solidão entre adultos mais velhos: evidências do Estudo Longitudinal da Saúde dos Idosos Brasileiros (ELSI-Brasil)

**DOI:** 10.1590/1980-549720240054.2

**Authors:** Karla Geovani Silva Marcelino, Luciana de Souza Braga, Maria Fernanda Lima-Costa, Juliana Lustosa Torres

**Affiliations:** I Universidade Federal de Minas Gerais – Belo Horizonte (MG), Brasil.; II Fundação Oswaldo Cruz, Núcleo de Estudos em Saúde Pública e Envelhecimento – Belo Horizonte (MG), Brasil.

**Keywords:** Características de residência, Características da família, Solidão, Envelhecimento, Saúde do idoso

## Abstract

**Objetivo:**

Examinar associação entre as características da família relativas ao cônjuge e aos(às) filhos(as) e a solidão em brasileiros com 50 anos e mais, considerando a ocorrência de solidão e seus níveis.

**Métodos:**

Estudo transversal, com dados de 7.163 participantes da segunda onda do Estudo Longitudinal da Saúde dos Idosos Brasileiros, um estudo nacionalmente representativo conduzido entre 2019–2021. Solidão foi mensurada por meio da escala de solidão da Universidade da Califórnia de três itens. As características da família consideraram: situação conjugal e moradia com cônjuge e presença de filhos(as) e moradia com eles. A análise estatística foi baseada em modelos de regressão do tipo *zero-inflated negative binomial*, um método estatístico que permite avaliar o desfecho de maneira dicotômica e como contagem.

**Resultados:**

Apenas as características da família relativas ao cônjuge se associaram à prevalência de solidão, independentemente de o cônjuge viver em outro domicílio (razão de prevalência — RP=0,35; intervalo de confiança de 95% — IC95% 0,23–0,53) ou no mesmo (RP=0,37; IC95% 0,30–0,45). Características familiares relativas ao cônjuge [cônjuge vivendo no mesmo domicílio (RP=0,80; IC95% 0,73–0,88)] e aos filhos [filhos vivendo em outro domicílio (RP=0,86; IC95% 0,77–0,95) ou no mesmo (RP=0,81; IC95% 0,72–0,92)] se associaram negativamente ao nível de solidão.

**Conclusão:**

As características da família contribuem para prevenir a solidão e para reduzir seus níveis. Serviços públicos de prestação de apoio social devem priorizar adultos mais velhos com núcleos familiares reduzidos.

## INTRODUÇÃO

A necessidade de se relacionar é uma característica humana, expressa por meio dos vínculos e dos sentimentos de companheirismo. Nesse sentido, a solidão é experimentada quando essa necessidade relacional, desejada, não é satisfeita, causando um sentimento desagradável^1,2^, o que difere de medidas objetivas de isolamento social^2^. Apesar de não haver consenso na literatura sobre a definição de solidão^2^, é bem descrito que ela pode gerar efeitos negativos na saúde mental e física dos indivíduos^2^, como declínio cognitivo^3,4^, aumento do risco de tentativas de suicídio^5^ e maior risco de doenças cardiovasculares^6^.

Além disso, a solidão é considerada uma epidemia^7^, com prevalência de 13,2% (intervalo de confiança de 95% — IC95% 9,2–18,6) entre pessoas de 60 anos ou mais, segundo dados de uma metanálise^8^. Estão sob maior risco de solidão adultos mais velhos sem um companheiro íntimo, aqueles que perderam seu companheiro recentemente, aqueles com uma rede social limitada e/ou com baixo nível de atividade social e aqueles deprimidos^9,10^.

Segundo Courtin e Knapp^11^, tanto a solidão quanto o isolamento social são problemáticos no envelhecimento em função da redução dos recursos econômicos e sociais, limitações funcionais, viuvez e mudanças nas estruturas familiares. A família é uma construção cultural e uma instituição social formada por no mínimo duas pessoas com laços ente elas, de parentesco, adoção ou conjugal, sendo a forma como se organiza no domicílio conhecida como arranjo domiciliar^12^.

Em relação aos arranjos domiciliares, residir com cônjuge e/ou filhos pode contribuir para que adultos mais velhos percebam ou recebam maior apoio social, emocional e instrumental^13^, o que pode contribuir para diminuir a solidão. Em estudo longitudinal conduzido na China entre pessoas com 65 anos e mais, a incidência de solidão foi semelhante entre aqueles que moravam com os filhos e aqueles que residiam apenas com o cônjuge, entretanto foi menor quando comparada à daqueles que moravam só^13^. Entre adultos mais velhos holandeses e italianos, de 55 a 89 anos, morar com o cônjuge, independentemente de coabitar com filhos, foi associado a menores níveis de solidão^14^. Estudo transversal conduzido com octogenários na Alemanha mostrou que ter um cônjuge morando em outro domicílio foi associado à solidão^10^.

Entre adultos mais velhos brasileiros com 50 anos e mais, morar sozinho^15,16^, ou morar com duas pessoas^16^, foi associado à solidão, contudo não foram encontrados estudos brasileiros que avaliaram a associação entre as características da família (ter cônjuge e/ou filhos e residir com eles) e solidão. No Brasil, a família é cultural e legalmente entendida como a principal responsável pelo bem-estar social, afetivo e econômico de seus membros^17^, o que pode diferir do contexto cultural de outros países e mostrar diferente papel em relação à solidão^18,19^. Desse modo, o objetivo do presente estudo foi examinar a associação entre as características da família [presença de cônjuge ou filhos(as) e coabitação com cônjuge ou filhos(as)] e a solidão, em brasileiros com 50 anos e mais, considerando tanto a ocorrência de solidão como seus diferentes níveis.

## MÉTODOS

Trata-se de estudo transversal realizado com base nos dados da onda 2 do Estudo Longitudinal da Saúde dos Idosos Brasileiros (ELSI-Brasil), conduzido entre 2019-2021. O ELSI-Brasil é um estudo representativo da população brasileira com 50 anos e mais residente na comunidade fundamentado em uma amostra probabilística. A amostra foi delineada em três estágios de seleção (municípios, setores censitários e domicílios) e inclui 70 municípios localizados nas cinco macrorregiões geográficas^20^. Nos domicílios selecionados, todos os moradores com 50 anos e mais foram elegíveis a participar. O questionário é composto de um módulo domiciliar, respondido por um morador do domicílio capaz de fornecer informações acerca das características do domicílio e informações socioeconômicas dos moradores, e de um módulo individual, respondido pelo próprio participante ou respondente substituto.

O ELSI-Brasil foi iniciado em 2015, e ondas subsequentes são realizadas a cada três anos, com reposições amostrais. Na onda 2, utilizada na presente análise, foram incluídos 9.949 participantes, com taxa de resposta de 75,9% em relação à onda anterior. A combinação de reposição amostral e participantes adicionais ao longo do tempo é importante para manter a representatividade nacional do estudo^20,21^. Mais detalhes podem ser consultados em outras publicações^20,21^ e na *homepage* da pesquisa (https://elsi.cpqrr.fiocruz.br/).

O ELSI-Brasil foi aprovado pelo Comitê de Ética em Pesquisa da Fundação Oswaldo Cruz (Minas Gerais), e o processo está cadastrado na Plataforma Brasil (Certificado de Apresentação para Apreciação Ética n^o^ 34649814.3.0000.5091). Os participantes assinaram termos de consentimento livre e esclarecido separados para cada um dos procedimentos da pesquisa.

Como o desfecho solidão é um evento subjetivo e depende da própria percepção do indivíduo^21^, foram elegíveis para a presente análise apenas os 9.108 participantes que responderam diretamente ao bloco psicossocial da entrevista (ou seja, não utilizaram respondente substituto no bloco S). Destes, incluíram-se apenas aqueles participantes que responderam tanto ao módulo domiciliar quanto ao módulo individual (n=7.301). Por último, excluíram-se os participantes com *missings* nas variáveis solidão (n=103) e características da família em relação ao cônjuge (n=0) e aos filhos (n=35). Portanto, a amostra final do presente estudo foi de 7.163 participantes, conforme Figura 1.

**Figura 1:** Fluxograma dos participantes incluídos no estudo. Estudo Longitudinal da Saúde dos Idosos Brasileiros (ELSI-Brasil), 2019–2021.

A solidão foi mensurada utilizando-se a escala de solidão da Universidade da Califórnia, Estados Unidos da América, de três itens^22^, composta de três perguntas relativas aos sentimentos de falta de companhia, sentir-se deixado de lado e sentir-se isolado das outras pessoas. As opções de resposta (nunca, algumas vezes ou sempre) geraram um escore final que variou de 3 (ausência de solidão) a 9 pontos, e a maior pontuação representa maior solidão. A escala em sua versão longa foi traduzida e validada para a população idosa brasileira, com um alpha de Cronbach de 0,88^23^. Os três itens incluídos na escala reduzida apresentaram correlações próximas a 0,60 em relação ao escore total (falta de companhia [r=0,60], sentir-se deixado de lado [r=0,54], sentir-se isolado de outras pessoas [r=0,61])^23^.

As variáveis independentes foram duas características da família, levando-se em conta a característica da família relativa ao cônjuge, pela presença deste, obtida no módulo individual, e a coabitação com cônjuge, obtida no módulo domiciliar. De modo semelhante, considerou-se a característica da família relativa aos filhos(as) obtida pela presença de filhos(as) e pela coabitação com eles(as).

As características da família relativas ao cônjuge se basearam na situação conjugal e no arranjo domiciliar. Aqueles que tinham um companheiro (casados, amasiados ou união estável) morando no domicílio foram incluídos no grupo "cônjuge vivendo no mesmo domicílio". Aqueles que relataram a presença de companheiro, mas reportaram que ele(a) não morava no domicílio, foram postos no grupo "cônjuge vivendo em outro domicílio". Os demais participantes, solteiros, divorciados ou viúvos, integraram o grupo "sem cônjuge".

As características da família relativas aos filhos(as) se basearam na presença de filhos(as) vivos(as) e no arranjo domiciliar. Aqueles que tinham pelo menos um(a) filho(a) vivo(a), mas nenhum deles(as) morando no domicílio, foram considerados no grupo "filhos(as) vivendo em outro domicílio". Aqueles que tinham pelo menos um(a) filho(a) vivo(a) e pelo menos um morando no domicílio foram incluídos no grupo "filhos(as) vivendo no mesmo domicílio". Os participantes que relataram não ter filhos(as) vivos foram para o grupo "sem filhos(as)".

Consideraram-se potenciais variáveis de confusão as características sociodemográficas e de saúde, de acordo com a literatura sobre fatores de risco para a solidão^9^, e que poderiam refletir em alterações das características da família. As variáveis sociodemográficas foram: sexo (feminino ou masculino); faixa etária (50–59; 60–69; 70–79; e ≥80 anos); e renda domiciliar *per capita*, em tercis (tercil inferior, tercil intermediário e tercil superior). As características de saúde foram: comprometimento cognitivo (não ou sim), depressão (não ou sim) e limitação em atividades básicas de vida diária (ABVDs) (não ou sim). O comprometimento cognitivo foi avaliado por testes cognitivos semelhantes aos utilizados no Health and Retirement Study^24^:

- orientação temporal em relação ao dia, mês, ano e dia da semana da entrevista, gerando um ponto para cada resposta correta;
- evocação imediata e tardia de dez palavras, na qual os participantes deveriam repetir dez palavras imediatamente após sua leitura (memória imediata) e repetir as mesmas palavras após 5 minutos da sua leitura (memória tardia). Foram computadas pontuações de 0 a 10 para o número de palavras recordadas nos testes de evocação imediata e tardia, de forma separada;
- fluência verbal semântica, avaliada pelo número de animais diferentes recordados em até 1 minuto.

Os resultados de cada um dos testes foram transformados em escores *z*, subtraindo-se o escore de cada participante do escore médio dos participantes e dividindo-o pelo desvio padrão (DP), para gerar um escore único obtido pela média dos escores *z* de todos os testes. O ponto de corte utilizado para definir o comprometimento cognitivo foi o escore *z* cognitivo global igual ou inferior a −1 DP^25^.

A depressão foi avaliada pelo autorrelato do diagnóstico médico. A limitação em ABVDs foi considerada quando o participante relatou dificuldade em pelo menos uma das seguintes atividades: caminhar de um cômodo a outro, vestir-se, tomar banho, usar o banheiro, comer, deitar-se na cama/levantar-se da cama.

Para a análise estatística, inicialmente, foi realizada a descrição das variáveis para a amostra total e segundo os níveis de solidão, por meio de proporções e médias. As diferenças observadas entre proporções foram analisadas por meio do teste χ^2^ de Pearson com correção de Rao-Scott, enquanto a diferença entre médias foi observada pelo teste de Wald ou teste t pareado. Posteriormente, foram construídos modelos de regressão do tipo *zero-inflated negative binomial* para estimar razões de prevalência (RP) e IC95% da associação entre as características da família e o escore de solidão. Esse modelo combina um modelo de excesso de zeros (ausência *versus* presença de solidão; escore 3 *versus* ≥4), que estima a RP da ausência de solidão, com um modelo de contagem do tipo binomial negativo (nível de solidão; escores variando de 4 a 9)^26^, que estima a RP média para cada aumento de uma unidade do escore de solidão. Esse tipo de modelo pôde ser adotado uma vez que o escore de solidão (variável dependente) apresentou sobredispersão (α=0.03; p<0.01) e se verificou um número elevado de pessoas sem solidão (escore 3; 56%)^27^.

O modelo *zero-inflated negative binomial* considera que a contagem zero (equivalente ao escore 3 de solidão) resulta de indivíduos que sempre relatam a ausência de solidão ("zeros estruturais") e de indivíduos expostos à solidão, mas que não a relataram no período do estudo ("zeros amostrais")^27^. Desse modo, para que o modelo considere o escore 3 como zero, o escore de solidão foi transformado para uma pontuação que varia de 0 a 6, entretanto os autores optaram por manter os escores originais (3 a 9) ao longo do texto, pois esses valores são reportados frequentemente na literatura. A fim de facilitar a interpretação das RPs no modelo que considera o excesso de zeros, os coeficientes foram invertidos para refletirem a RP da prevalência de solidão (escore 3 *versus* ≥4), e não a ausência de solidão (excesso de zeros).

Todas as análises foram realizadas no *software* Stata/Se (StataCorp., College Station, TX, Estados Unidos), versão 17.0, considerando-se o delineamento amostral e os pesos dos participantes.

## RESULTADOS

Entre os 7.163 participantes incluídos, a média de idade foi de 63,1 anos (DP=8,8 anos). Destes, 44,1% foram classificados com solidão leve, perfazendo um escore médio de 5,2 (DP=1,2) e mediana de 5 (amplitude interquartil de 4 a 6) quando a solidão estava presente. Como a média e a mediana foram semelhantes e a média conseguiu mostrar mais as diferenças entre os grupos analisados, utilizaram-se as médias na descrição das variáveis.

A Tabela 1 mostra as características dos participantes, total e segundo a proporção e média do escore de solidão. A maioria dos participantes era do sexo feminino (52,5%), com idade até 69 anos (77,6%), 54% viviam com cônjuge no mesmo domicílio e 58% tinham filhos vivendo em outro domicílio. As características da família variaram segundo a prevalência de solidão (escore ≥4) apenas quando se considerou o cônjuge (p<0,001), entretanto as médias do nível de solidão (escore de 4 a 9) foram diferentes tanto entre as características da família em relação ao cônjuge e quanto entre as características em relação aos filhos(as), sendo menores nas categorias cônjuge vivendo no mesmo domicílio (p<0,001), filhos(as) vivendo em outro domicílio (p=0,006) e filhos(as) vivendo no mesmo domicílio (p<0,001).

**Tabela 1:** Distribuição das características sociodemográficas, de saúde e da família dos participantes, total e segundo o nível de solidão (Estudo Longitudinal da Saúde dos Idosos Brasileiros, 2019–2021).

Variáveis		Total (%)	Solidão		
			Nenhuma solidão (escore 3) (% e IC95%)*	Presença de solidão (escore ≥4) (% e IC95%)*	Nível de solidão (escore de 4 a 9) (média e SD)†
Sociodemográficas					
Sexo					
	Feminino	52,5	**49,1 (45,0–53,2)**	**50,9 (46,8–55,0)**	5,3 (1,3)
	Masculino	47,5	**63,3 (58,8–67,5)**	**36,7 (32,5–41,2)**	5,2 (1,1)
Faixa etária (anos)					
	50–59	47,7	55,6 (58,9–60,2)	44,4 (39,8–49,1)	**5,3 (1,0)**
	60–69	29,9	57,0 (53,2–60,8)	43,0 (39,2–46,8)	**5,1 (1,3)**
	70–79	16,1	56,5 (52,2–60,7)	43,5 (39,3–47,8)	**5,2 (1,5)**
	≥80	6,3	50,6 (43,8–57,5)	49,4 (42,5–56,2)	**5,3 (1,5)**
Renda domiciliar *per capita*					
	Tercil inferior	33,4	54,2 (48,6–59,8)	45,8 (40,2–51,4)	**5,4 (1,2)**
	Tercil intermediário	33,3	53,5 (49,0–58,0)	46,5 (42,0–51,0)	**5,2 (1,3)**
	Tercil superior	33,3	59,1 (54,9–63,2)	40,9 (36,8–45,1)	**5,1 (1,2)**
Saúde					
Comprometimento cognitivo‡					
	Não	78,4	**57,5 (53,4–61,6)**	**42,5 (38,4-46,6)**	**5,1 (1,2)**
	Sim	21,6	**52,1 (47,5–56,6)**	**47,9 (43,4–52,5)**	**5,4 (1,4)**
Depressão					
	Não	87,3	**58,7 (54,6–62,7)**	**41,3 (37,3–45,4)**	**5,1 (1,2)**
	Sim	12,7	**36,4 (31,1–42,1)**	**63,6 (57,9–68,9)**	**5,8 (1,4)**
Limitação em ABVDs§					
	Não	91,9	**57,6 (53,7–61,4)**	**42,4 (38,6–46,3)**	**5,2 (1,2)**
	Sim	8,1	**35,9 (30,0–42,3)**	**64,1 (57,7–70,0)**	**5,6 (1,3)**
Características da família					
Cônjuge					
	Sem cônjuge	42,0	**42,1 (38,4–45,9)**	**57,9 (54,1–61,6)**	**5,4 (1,4)**
	Cônjuge vivendo em outro domicílio	4,1	**63,2 (53,7–71,8)**	**36,8 (28,2–46,3)**	**5,4 (1,2)**
	Cônjuge vivendo no mesmo domicílio	53,9	**66,0 (61,2–70,5)**	**34,0 (29,5–38,8)**	**5,0 (1,0)**
Filhos(as)					
	Sem filhos(as)	9,1	50,2 (42,5–57,9)	49,8 (42,1–57,5)	**5,5 (1,1)**
	Filhos(as) vivendo em outro domicílio	57,9	55,5 (52,0–59,0)	44,5 (41,0–48,0)	**5,2 (1,3)**
	Filhos(as) vivendo no mesmo domicílio	33,0	57,9 (52,1–63,5)	42,1 (36,5–47,9)	**5,1 (1,2)**
**N total**		7.163	3.823	2.897	443

* valores em negrito: p<0,05 baseado no teste χ^2^ de Pearson com correção de Rao-Scott;

† valores em negrito: p<0,05 baseado no teste de Wald ou teste t pareado;

‡ considerando orientação temporal, memórias imediata e tardia e fluência verbal semântica;

§ dificuldade em caminhar de um cômodo a outro, vestir-se, tomar banho, usar o banheiro, comer ou deitar-se na cama/levantar-se da cama. IC95%: intervalo de confiança de 95%; SD: desvio padrão; ABVDs: atividades básicas da vida diária.

A Tabela 2 descreve a associação entre a prevalência/nível de solidão e as características sociodemográficas, de saúde e da família. Associaram-se a menores prevalências de solidão as características sociodemográficas (sexo masculino), enquanto as características de saúde se associaram a maiores prevalências de solidão (depressão e limitação em ABVDs). No que tange às características da família, a prevalência de solidão foi 65% menor entre aqueles que tinham cônjuge vivendo em outro domicílio (RP=0,35; IC95% 0,23–0,53) e 63% menor entre adultos mais velhos cujos cônjuges viviam no mesmo domicílio (RP=0,37; IC95% 0,30–0,45), em comparação àqueles sem cônjuge.

**Tabela 2:** Resultados dos modelos brutos e ajustado da associação entre características da família e solidão, ajustada por características sociodemográficas e de saúde (n=6.063) (Estudo Longitudinal da Saúde dos Idosos Brasileiros, 2019–2021)*.

Variáveis		Presença de solidão (escore 3 versus ≥4)				Nível de solidão (aumento de uma unidade no escore de 4 a 9)			
		Modelos brutos		Modelo ajustado		Modelos brutos		Modelo ajustado	
		RP†	IC95%	RP†	IC95%	RP	IC95%	RP	IC95%
Características sociodemográficas									
Sexo masculino (*versus*feminino)		**0,56**	**0,48–0,65**	**0,75**	**0,61–0,92**	0,95	0,88-1,02	1,02	0,93–1,11
Faixa etária (*versus* 50–59 anos)									
	60–69	0,96	0,81–1,12	0,92	0,74–1,14	**0,89**	**0,83–0,97**	0,91	0,84–1,00
	70–79	0,98	0,81–1,18	0,83	0,63–1,07	0,93	0,85–1,02	**0,88**	**0,87–0,97**
	≥80	1,17	0,91–1,51	0,95	0,64–1,40	1,02	0,92–1,13	0,96	0,82–1,12
Renda domiciliar *per capita* (*versus* tercil inferior)									
	Tercil intermediário	1,09	0,89–1,34	1,06	0,83–1,36	0,93	0,84–1,02	**0,90**	**0,81–0,99**
	Tercil superior	0,88	0,72–1,08	0,80	0,62–1,01	**0,86**	**0,78–0,95**	**0,82**	**0,74–0,91**
Características de saúde									
	Comprometimento cognitivo (*versus* não)	**1,13**	**1,05–1,23**	1,00	0,80–1,25	1,16	0,98–1,38	1,07	0,98–1,17
	Depressão (*versus* não)	**2,17**	**1,76–2,67**	**2,05**	**1,52–2,77**	**1,44**	**1,34–1,56**	**1,42**	**1,28–1,56**
	Limitação em ABVDs (*versus* não)	**2,44**	**1,87–3,20**	**1,92**	**1,31–2,80**	**1,27**	**1,16–1,39**	**1,18**	**1,04–1,34**
Características da família									
Cônjuge (*versus* sem cônjuge)									
	Cônjuge vivendo em outro domicílio	**0,36**	**0,26–0,51**	**0,35**	**0,23–0,53**	1,01	0,87–1,17	1,01	0,84–1,22
	Cônjuge vivendo no mesmo domicílio	**0,39**	**0,33–0,45**	**0,37**	**0,30–0,45**	**0,83**	**0,77–0,89**	**0,80**	**0,73–0,88**
Filhos(as) [*versus* sem filhos(as)]									
	Filhos(as) vivendo em outro domicílio	0,87	0,67–1,12	1,29	0,92–1,81	**0,86**	**0,78–0,95**	**0,86**	**0,77–0,95**
	Filhos(as) vivendo no mesmo domicílio	0,84	0,63–1,13	1,18	0,81–1,73	**0,80**	**0,71–0,90**	**0,81**	**0,72–0,92**

ABVDs: atividades básicas da vida diária; RP: razão de prevalência; IC95%: intervalo de confiança de 95%;

* valores em negrito: p<0,05 baseado no *zero-inflated negative binomial regression*;

† RP invertidas de modo a refletirem a presença de solidão (escore ≥4), e não a ausência de solidão (escore=3).

Referente ao nível de solidão, a Tabela 2 mostra que se associaram a menores níveis de solidão as características sociodemográficas (faixa etária [70–79 anos] e renda domiciliar *per capita* [tercil intermediário e superior]), enquanto as características de saúde se associaram a maiores níveis de solidão (depressão e limitação em ABVDs). Em relação às características da família, o nível de solidão foi 20% menor, em média, entre adultos mais velhos cujo cônjuge vive no mesmo domicílio (RP=0,80; IC95% 0,73–0,88), em comparação ao das pessoas que não têm cônjuge. O mesmo não foi observado para adultos mais velhos cujo cônjuge vive em outro domicílio (RP=1,01; IC95% 0,84–1,22). Quanto aos(as) filhos(as), o nível de solidão foi em média 14% menor entre aqueles cujos(as) filhos(as) vivem em outro domicílio (RP=0,86; IC95% 0,77–0,95) e 19% menor entre aqueles cujos(as) filhos(as) vivem no mesmo domicílio (RP=0,81; IC95% 0,72–0,92), quando comparado ao das pessoas que não têm filhos(as).

## DISCUSSÃO

Este estudo mostrou que as características da família estiveram associadas à solidão tanto em relação à prevalência de solidão quanto ao maior nível de solidão. Enquanto as características da família relativas ao cônjuge se associaram à ocorrência de solidão (pessoas com cônjuge morando no mesmo domicílio ou em outro domicílio apresentam menores prevalências de solidão), ter filhos vivendo no mesmo domicílio ou em outro domicílio não foi significativo. Adicionalmente, uma vez que a pessoa sinta solidão, as características da família relativas ao cônjuge e aos(as) filhos(as) se associaram inversamente ao maior nível de solidão. Menor nível de solidão foi observado entre adultos mais velhos que viviam com cônjuge no mesmo domicílio e entre aquelas pessoas com filhos(as), morando com eles(as) ou não.

Nossos achados corroboram a literatura existente de que a maior prevalência de solidão foi encontrada entre aqueles adultos mais velhos sem cônjuge^9,10,28–30^. Esses achados podem ser explicados pela teoria da seletividade socioemocional proposta por Carstensen^31^, em que as perdas socioemocionais próprias do envelhecimento são compensadas pela autorregulação emocional, ou seja, os recursos socioemocionais passam a ser redistribuídos em decorrência das mudanças na perspectiva de tempo futuro, e relacionamentos próximos se tornam mais gratificantes. Outra explicação pode ser a influência que a cultura exerce na experiência da solidão, moderando o efeito de diferentes tipos de relacionamento na solidão, conforme demonstrado no estudo de Lykes e Kemmelmeier. Em países europeus coletivistas, o sentimento de pertencimento é muito importante para os indivíduos, uma vez que estes dão demasiada atenção às relações familiares e aos laços afetuosos^19^.

Considerando a amostra do ELSI-Brasil, estudo transversal prévio mostrou que pessoas vivendo em arranjos de moradia de duas ou de três pessoas ou mais apresentavam praticamente a mesma prevalência de solidão algumas vezes (31,1 e 32,5, respectivamente)^16^. Sabe-se que no Brasil o tipo de arranjo domiciliar mais frequente, incluindo duas pessoas, é a coabitação com cônjuge, apesar das novas configurações dos arranjos domiciliares^32^.

O cônjuge, muitas vezes, pode ser a pessoa mais próxima a fornecer apoio social emocional positivo^33^, contribuindo para a diminuição de estressores associados a pior saúde mental^34^ e ampliando as oportunidades de envolvimento em atividades relacionadas ao companheiro, o que facilita a participação social e a formação de vínculos^35^. Nesse sentido, estudo longitudinal com mexicanos de 50 anos e mais mostrou que pessoas com cônjuge que sentiam apoio do parceiro tiveram níveis mais baixos de solidão após três anos^36^.

Quanto às características da família, o presente estudo mostrou que a presença de cônjuge vivendo em outro domicílio não se associou ao maior nível de solidão, diferentemente do encontrado previamente em octogenários na Alemanha^10^. Em primeiro lugar, ter um cônjuge que mora em outro domicílio pode ser um marco de segunda união, experiências negativas com o cônjuge anterior ou a própria institucionalização^10,37^. Segundo, o cônjuge pode fornecer apoio social mesmo que não viva no mesmo domicílio e apresente certa distância física^19^. Sabe-se que o equilíbrio e a reciprocidade na interação social são importantes^38^, e residir em domicílios diferenciados pode ser um fator importante para a preservação do equilíbrio e reciprocidade entre o casal, entretanto a literatura ainda é limitada em relação à existência de cônjuge vivendo em outro domicílio, dificultando a discussão desses resultados.

No que tange aos filhos, os achados deste estudo revelam que ter filhos vivendo no mesmo domicílio ou em outro reduz os níveis de solidão. Uma possível explicação para níveis mais baixos de solidão entre aqueles com filhos pode ser, além do aumento da rede familiar, a diversidade de contato com filhos, netos e outros parentes^10^, independentemente da possível distância geográfica entre eles, que pode vir a ser intermediada por contatos virtuais e presenciais.

No Brasil, o contato presencial ou virtual com filhos que moram em outro domicílio é muito frequente entre adultos mais velhos^39^, sendo por si só uma importante fonte de apoio social emocional e instrumental em caso de necessidades^39,40^. No Japão, estudo longitudinal entre indivíduos com 65 anos e mais encontrou menor probabilidade de relatar solidão entre aqueles que residiam com pelo menos um dos filhos em comparação com aqueles que não residiam com nenhum filho^41^. Para aqueles que não tinham nenhum filho residindo com eles, ter um número maior de filhos foi relacionado à menor probabilidade de relatar solidão^41^.

Em estudo de Zoutewelle-Terovan e Liefbroer com amostra representativa de indivíduos com idade entre 50 e 85 anos de 12 nações europeias, níveis mais altos de solidão foram encontrados entre aqueles que não tinham filhos, e a magnitude do efeito diversificou-se de acordo com o valor cultural do país^42^. O fato de ter filho pode gerar nos indivíduos uma sensação de segurança mútua, até mesmo porque, em sociedades coletivistas, a relação familiar é fundamental para o bem-estar^19^.

A literatura aponta que aqueles que não possuem filhos tendem a diversificar mais as suas fontes de apoio social, assim como ocorre com casais com baixo apoio social mútuo^43^, o que pode compensar o sentimento de solidão, contudo profissionais da saúde e assistência social devem ficar atentos aos sentimentos dos adultos mais velhos e às características da família com vistas à manutenção de relacionamentos significativos, prevenção e redução de risco para solidão.

Este estudo apresenta pontos fortes e fracos. Como ponto forte, destaca-se o pioneirismo do estudo ao analisar as características familiares relacionadas à situação conjugal, à presença de filhos(as) e ao arranjo domiciliar de forma conjunta, bem como a solidão em uma amostra representativa de brasileiros mais velhos. Além disso, o uso do modelo *zero-inflated negative binomial* nas análises estatísticas permite distinguir os fatores associados à prevalência de solidão e a seu nível, contudo a escala empregada para avaliar a solidão, apesar de amplamente utilizada internacionalmente, não foi validada em sua versão curta para a população brasileira. Ainda, o caráter transversal do estudo não permite estabelecer relações de causalidade entre as características familiares e a solidão. Desse modo, estudos futuros poderão avaliar a direcionalidade das associações encontradas. Vale ressaltar que o ELSI-Brasil é um estudo de coorte prospectiva e que futuras análises longitudinais poderão investigar a temporalidade das associações encontradas no presente estudo.

Além disso, salienta-se que as características da família são importantes tanto para prevenir a solidão quanto para reduzir seus níveis, de maneira que ações assistenciais devem ser direcionadas aos adultos mais velhos com núcleos que proporcionem menor apoio familiar, como na ausência de cônjuge e filhos(as). Sugere-se, portanto, investir na oferta de serviços públicos que garantam assistência centrada na necessidade dos indivíduos, com maior apoio social provido pelo Estado às pessoas com núcleos familiares menores. A solidão é um sentimento negativo considerado como problema de saúde pública e é potencialmente modificável, sendo fundamental seu rastreio, principalmente entre aqueles em maior risco.
